# NTPase and 5′-RNA Triphosphatase Activities of Chikungunya Virus nsP2 Protein

**DOI:** 10.1371/journal.pone.0022336

**Published:** 2011-07-19

**Authors:** Yogesh A. Karpe, Pankaj P. Aher, Kavita S. Lole

**Affiliations:** Hepatitis Division, National Institute of Virology, Microbial Containment Complex, Pashan, Pune, India; Agency for Science, Technology and Research - Singapore Immunology Network, Singapore

## Abstract

Chikungunya virus (CHIKV) is an insect borne virus (genus: *Alphavirus*) which causes acute febrile illness in humans followed by a prolonged arthralgic disease that affects the joints of the extremities. Re-emergence of the virus in the form of outbreaks in last 6–7 years has posed a serious public health problem. CHIKV has a positive sense single stranded RNA genome of about 12,000 nt. Open reading frame 1 of the viral genome encodes a polyprotein precursor, nsP1234, which is processed further into different non structural proteins (nsP1, nsP2, nsP3 and nsP4). Sequence based analyses have shown helicase domain at the N-terminus and protease domain at C-terminus of nsP2. A detailed biochemical analysis of NTPase/RNA helicase and 5′-RNA phosphatase activities of recombinant CHIKV-nsP2T protein (containing conserved NTPase/helicase motifs in the N-terminus and partial papain like protease domain at the C-terminus) was carried out. The protein could hydrolyze all NTPs except dTTP and showed better efficiency for ATP, dATP, GTP and dGTP hydrolysis. ATP was the most preferred substrate by the enzyme. CHIKV-nsP2T also showed 5′-triphosphatase (RTPase) activity that specifically removes the γ-phosphate from the 5′ end of RNA. Both NTPase and RTPase activities of the protein were completely dependent on Mg^2+^ ions. RTPase activity was inhibited by ATP showing sharing of the binding motif by NTP and RNA. Both enzymatic activities were drastically reduced by mutations in the NTP binding motif (GKT) and co-factor, Mg^2+^ ion binding motif (DEXX) suggesting that they have a common catalytic site.

## Introduction

Chikungunya virus (CHIKV) (family *Togaviridae,* genus *Alphavirus*) is transmitted by *Aedes* mosquitoes. It causes an acute febrile illness associated with severe joint pain that can persist for a long time even after viral clearance. Due to changing patterns of vector distribution, abundance in response to climate change and increased vector-human contact, CHIKV is regarded as a potential worldwide public health problem, with no preventive or therapeutic means available.

CHIKV is enveloped, single stranded positive sense RNA virus having genome of≈12,000 nt, encoding four non-structural (ns1–4) and three main structural proteins (C, E2 and E1) with organization as: 5′-cap-(non-structural proteins)-(junction region)-(structural proteins)-Poly (A) tail-3′. Prototype viruses like Sindbis Virus (SINV) and Semliki Forest Virus (SFV) of this family have been extensively studied. ORF1 encodes a polyprotein precursor termed as nsP1234 and processed into different non structural proteins (nsP1, nsP2, nsP3 and nsP4) after stepwise proteolytic cleavages. The 3′-terminal one-third of the genome encodes for viral structural polyprotein which is expressed from a separate subgenomic mRNA and cleaved co-translationally and posttranslationally into structural proteins C, E1, E2 [1]. Alphavirus nsP4, the viral RNA-dependent RNA polymerase (RdRp) and the processing intermediates or mature products of nsP123 are essential components of the viral RNA replication and transcription complexes [2].

Alphaviral nsP2 is a multifunctional protein [3], [4], [5], [6]. Nucleoside triphosphatase, helicase, and RNA-dependent 5′- triphosphatase activities have been located in the N-terminus of the protein while the proteolytic domain has been mapped to its C-terminal part [3], [7]. It forms a papain-like thiol protease. The nsP2 protease is responsible for cleavages in the non-structural polyprotein [8]; [9]. CHIKV protease activity of nsP2 has been demonstrated [10] however enzymatic activities associated with N-terminus have not been shown as yet.

Helicase seems to be essential for the function of viral RdRp in positive sense RNA viruses [11]. In addition, it may be involved in capping, RNA translocation, genome packaging, protection of RNA at replication center, modulating RNA-protein interactions etc. CHIKV nsP2 helicase belongs to the superfamily 1 (SF1) and shows seven conserved signature motifs (I, Ia, II, III, IV, V and VI) which form the core of the enzyme.

In this study we carried out detailed biochemical analysis of NTPase/RNA helicase and 5′-RNA phosphatase activities of truncated CHIKV nsP2 containing partial papain like protease domain at the C-terminus and the conserved NTPase/helicase motifs in the N-terminus of the protein.

## Results

### Expression and purification of CHIKV-nsP2T and its mutants

**Figure 1:**
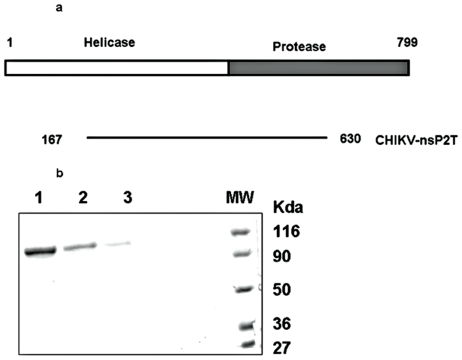
Expression of truncated CHIKV nsP2. Schematic representation of CHIKV nsP2: N- terminal helicase domain (white) and C-terminal protease domain (gray) are indicated in the figure. The two proteins expressed were, CHIKV-Hel (166–441 a.a.) and CHIKV-nsP2T (166–630 a.a.). Both this proteins spanned all seven signature motifs of SF1 helicases which are conserved among Alphaviruses. SDS-PAGE analysis: HPLC purified proteins were analyzed on 10% SDS-PAGE. Lanes are: (1) Wild type CHIKV-nsP2T, (2) nsP2T-mut I, (3) nsP2T-mut II, (MW) Molecular weight marker.

In order to locate the exact termini of nsP2 in the CHIKV genome and to design the primers for amplification, SFV and SINV sequences were aligned with the CHIKV sequence using Multalign program. Different motifs of the enzymes were identified in the same way using amino acid alignments (Figure 1a) (Table 1). Truncated CHIKV nsP2 (166–630 a.a. of nsP2, 465 amino acids) was expressed as a fusion protein with N-terminal Maltose-binding protein in bacterial expression system and purified using amylose affinity chromatography with native buffer system. Protein was further purified by gel filtration chromatography using HPLC. The purified protein showed single band of size ∼95 kDa (Figure 1b).

**Table 1:** Helicase sequence motifs of the CHIKV-nsP2 identified by using amino acid alignments.

Motif	Amino acid position in CHIKV nsP2	Motif Sequence
I	180–198	VIGVFGVPGSGKSAIIKN
IA	203–217	DLVTSGKKENCQEI
II	247–256	VLYVDEAFA
III	276–283	LCGDPKQ
IV	304–313	YHKSISRRC
V	373–394	YEVMTAAASQGLTRKGVYAVR
VI	405–419	TSEHVNVLLTRTEG

Two mutants of the CHIKV nsP2 (nsP2 mut I and nsP2 mut II) were generated by site directed mutagenesis. These proteins were also expressed as MBP tagged proteins in E.coli and purified using the same protocol as that used for the wild type protein. In nsP2 mut I, conserved lysine residue in motif I/Walker A motif (GKS), which is known to have crucial role in NTP binding was mutated to alanine (K to A). In nsP2 mut II, conserved aspartic acid and glutamic acid residues from motif II/Walker B motif (DEAF), known to be involved in Mg^+2^ binding, were modified to alanine-alanine (DE to AA). Both proteins showed same sized bands on SDS-PAGE similar to wild type protein (Figure 1b). Proteins were also confirmed by western blot analysis using anti-MBP antibodies (data not shown).

### ATPase activity of CHIKV-nsP2T

NTP hydrolysis is known to provide energy required for the motor activity and translocation of helicases on single stranded RNA. The NTPase activity of CHIKV helicase was quantified using a sensitive colorimetric assay that measures the total amount of orthophosphate released, based on the colored complex of phosphomolybdate and malachite green. The assays were performed in 50 µl reaction volumes, using standard 96-well plate.

**Figure 2:**
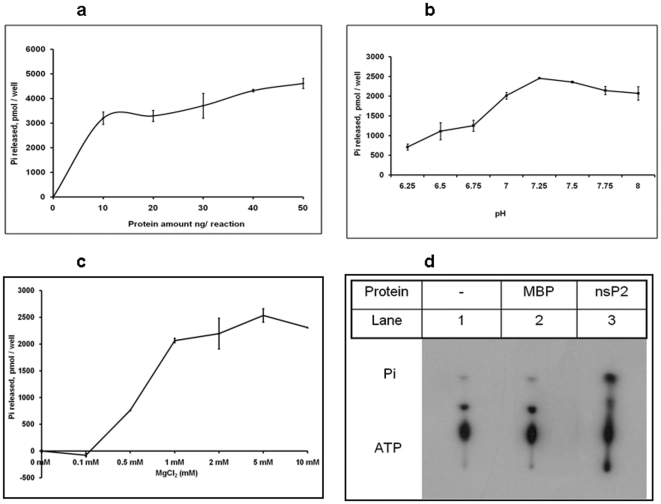
ATPase activity of CHIKV-nsP2T. At different enzyme concentrations: CHIKV-nsP2T protein was incubated at different concentrations (5 ng to 50 ng) in a 50 µl reaction containing 50 mM MOPS at pH 7.25, 1 mM ATP, 1 mM MgCl_2,_ at 37°C for 30 min. Released phosphate was quantitated as described in the experimental procedures. Effect of pH on ATPase activity: CHIKV-nsP2T protein was incubated in a 50 µl reaction containing 50 mM MOPS at pH 6.25–8.0, 1 mM ATP, 1 mM MgCl_2,_ at 37°C for 30 min. Released phosphate was quantitated as described in the experimental procedures. Effect of MgCl_2_ concentration on ATPase activity: CHIKV-nsP2T protein was incubated in a 50 µl reaction containing 50 mM MOPS at pH 7.25, 0–5 mM MgCl_2_, 1 mM ATP, at 37°C for 30 min. Released phosphate was quantitated as described in the experimental procedures. Analysis of released phosphate on TLC: Reaction was carried out in 20 µl containing 1 nM CHIKV-nsP2T, 50 mM MOPS (pH 7.25), 1 mM MgCl_2_, 1 mM ATP, 1 µCi of [γ-^32^P] ATP, incubated at 37°C for 30 min and 1 µl of the mixture was analyzed by TLC and processed for autoradiography.

Initially, ATPase activity of the CHIKV-nsP2T was carried out at different protein concentrations (5–50 ng/reaction), MgCl_2_ concentrations (0–10 mM) and at different pH (6.25–8.0) to optimize the NTPase assay conditions. There was a gradual increase in the released phosphate with increase in the protein concentration from 10–50 ng/reaction (Figure 2a). Enzyme showed similar activity in 7.0–8.0 pH range, with a minor peak at pH 7.25 (Figure 2b). There was a gradual enhancement in the ATPase activity with increasing MgCl_2_ concentrations from 0.1 to 1 mM. Released phosphate levels did not increase significantly with further increase in the Mg^2+^ ion concentrations (Figure 2c). All further NTPase reactions were carried out at pH 7.25 in presence of 1 mM MgCl_2_. Optimum temperature of the reaction was 37°C (data not shown). ATPase activity of the CHIKV-nsP2T was absolutely dependent on Mg^2+^, as no detectable ATPase activity was observed without MgCl_2_. There was no Pi release seen when maltose binding protein alone was incubated with γ-^32^P labeled ATP (Figure 2d).

**Figure 3:**
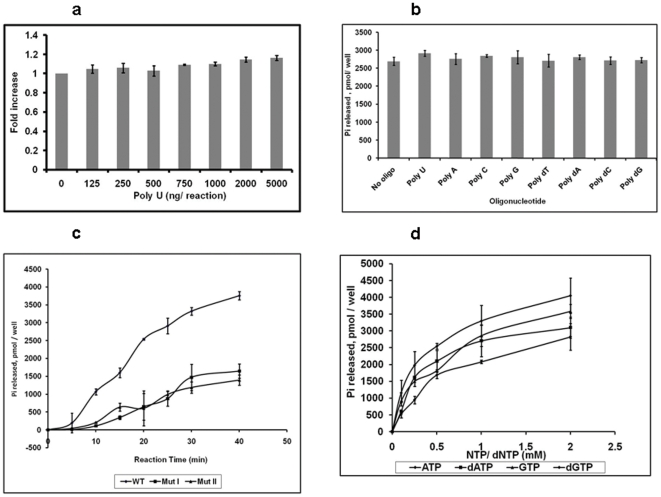
NTPase activity of CHIKV-nsP2T. Effect of poly (U) RNA on ATPase activity: CHIKV-nsP2T protein was incubated in a 50 µl reaction containing 50 mM MOPS at pH 7.25, 1 mM ATP, 1 mM MgCl_2_, poly (U) RNA (0–5000 ng), at 37°C for 30 min. Released phosphate was quantitated as described in the experimental procedures. Effect of different nucleic acid oligonucleotides on ATPase activity: CHIKV-nsP2T protein was incubated in a 50 µl reaction containing 50 mM MOPS at pH 7.25, 1 mM ATP, 1 mM MgCl_2_, and different homopolynucleotides (25 ng/µl of the reaction), at 37°C for 30 min. Released phosphate was quantitated as described in the experimental procedures. ATPase activity of CHIKV-nsP2T mutant proteins (mut I and mut II): CHIKV-nsP2T mutant proteins were incubated in a 50 µl reaction containing 50 mM MOPS at pH 7.25, 1 mM MgCl_2_, 1 mM ATP, at 37°C for 30 min. Released phosphate was quantitated as given in the experimental procedures. NTPase activity of CHIKV-nsP2T: CHIKV-nsP2T protein was incubated in a 50 µl reaction containing 50mM MOPS at pH 7.25, 1 mM MgCl_2_, and increasing concentrations of different NTPs, at 37°C for 30 min. Released phosphate was quantitated as described in the experimental procedures.

NTPase activity of helicase-like protein is typically stimulated by nucleic acids, particularly by poly (U). To test that, different concentrations of Poly (U) (125 ng to 5000 ng/reaction) were added and released phosphate was measured. There was no increase in the activity of CHIKV-nsP2T in presence of poly (U). Amount of phosphate released remained same even after increasing the oligo concentration up to 5.0 µg/reaction (Figure 3a). Results remained same even when increased amount of the protein was added in the reaction (10 nM, 50 ng/reaction) (data not shown). Similarly, there was no enhancement observed in presence of any of the other RNA/DNA homopolynucleotides (polyA/U/G/C/dA/dT/dG-/dC) (Figure 3b).

Walker A and Walker B motifs are highly conserved sequence motifs in NTP binding proteins. It was of interest to examine the role of these motifs in the ATPase activity of the CHIKV-nsP2T. Two mutant proteins- CHIKV nsP2 mut I (Walker A mutant) and nsP2 mut II (Walker B mutant) were assayed for their ATPase activities at the optimized reaction conditions as mentioned above. Both mutant proteins showed fivefold reduction in the activity (Figure 3c). This also proved that CHIKV-nsP2T NTPase activities obtained in the present study were actual activities of the purified recombinant proteins and not that of contaminating E.coli proteins.

### Comparison of NTP substrates in the NTPase reaction

The specificity of hydrolysis of NTPs by CHIKV-nsP2T was examined using four common NTPs and dNTPs as substrates. There was significant level of hydrolysis of ATP, dATP, GTP and dGTP (Figure 3d), low level hydrolysis of CTP, dCTP and UTP and no hydrolysis of dTTP (data not shown). The kinetic parameters *k_cat_* (turnover number) and *K_m_* (Michaelis constant) were determined from Lineweaver-Burk plots of the enzyme activity for different concentrations of ATP, dATP, GTP and dGTP. The *k_cat_/K_m_* value was calculated for each NTP which is a measure of the overall activity of the enzyme for that substrate, Table 2. ATP was the best substrate for CHIKV-nsP2T showing the highest turnover (30.87 min^−1^) and *k_cat_/K_m_* value (1.72 mM^−1^min^−1^). Kinetic parameters were not determined for CTP, dCTP and UTP and dTTP. Taken together, the data suggest that CHIKV-nsP2T prefers ATP as energy source but if required can also utilize dATP, GTP and dGTP as substrates.

**Table 2:** Analysis of CHIKV-nsP2 NTPase substrate specificity.

Nucleotide	Km (nM)	Kcat (min^−1^)	Kcat/Km (min^−1^ nM^−1^)
ATP	17.9	30.87	1.72
dATP	18.8	24.74	1.31
GTP	29.1	30.22	1.03
dGTP	33.9	24.75	0.73

### RNA/DNA strand displacement activities

**Figure 4:**
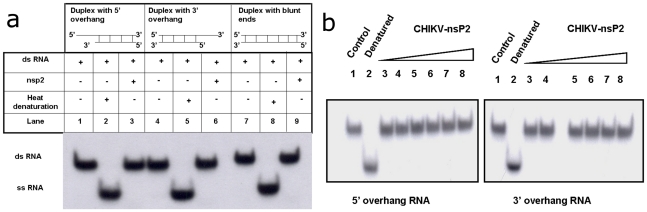
Strand displacement activity of CHIKV-nsP2T. With different RNA substrates: Unwinding activity of the protein was checked using different RNA substrates. CHIKV-nsP2 protein was incubated with RNA duplexes with 5′ overhang (lanes 1, 2, 3); with 3′ overhang (lanes 4, 5, 6) and with blunt ends (7, 8, and 9). With different protein concentrations: Unwinding activity was carried out in presence of increasing concentrations of CHIKV-nsP2T using RNA substrate with both 5′ and 3′ overhangs, Lanes (1) control, (2) heat denatured substrate RNA, and (3 to 8) different CHIKV-nsP2T concentrations (1, 10, 50, 100, 500 and 1000 ng).

To characterize unwinding activity of CHIKV-nsP2T, RNA/DNA duplexes with either 3′- or 5′-single stranded overhangs or duplexes with blunt ends were used. The blunt end duplexes were generated by annealing 28 nt oligonucleotides while for duplexes with 5′ and 3′ overhangs, 28 nt and 16 nt long oligonucleotides were used and both had 12 nucleotide single stranded stretches at respective ends [12]. One strand in each of the three RNA or DNA duplexes was 5′-end labeled. Duplex unwinding assays were carried out at similar conditions which were optimized for the ATPase activity. There was no detectable strand displacement by CHIKV-nsP2T with any of the RNA/DNA duplexes with 1 nM protein/reaction (Figure 4a). Further, unwinding assays were carried out using different concentrations of the enzyme from 1 ng-1000 ng/reaction. There was no visible unwinding activity seen even at higher concentrations of enzyme in the reactions (Figure 4b).

### RNA-5′-triphosphatase activity

**Figure 5:**
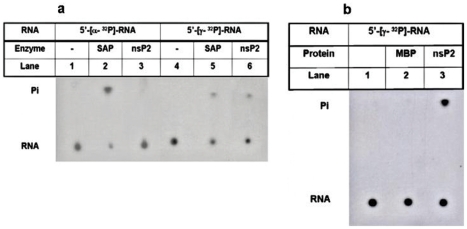
RNA 5′-triphosphatase activity of CHIKV-nsP2T. MBP-nsP2T and shrimp alkaline phosphatase (SAP) were incubated with 5′-γ-^32^P/5′-α-^32^P labeled nonspecific RNA (5′-GGGA_24_-3′) substrates separately at 37°C for 30 min and analyzed by TLC. Lanes 1) 5′-[α-^32^P]-RNA, 2) 5′-[α-^32^P]-RNA with SAP, 3) 5′-[α-^32^P]-RNA with CHIKV-nsP2T, 4) 5′-[γ-^32^P]-RNA, 5) 5′-[γ-^32^P]-RNA with SAP, 6) 5′-[γ-^32^P]-RNA with CHIKV-nsP2T. CHIKV-nsP2T was incubated with 5′-[γ-^32^P] labeled CHIKV 5′-NCR RNA at 37°C for 30 min, products was analyzed by TLC and the plate was exposed to X-ray film. Lanes 1) RNA without protein, 2) RNA with MBP, 3) RNA with CHIKV-nsP2T.

For RNA triphosphatase activity studies, 5′-[γ-^32^P]-RNA or 5′-[α-^32^P]-RNA substrates either with non-specific sequence-(5′-GGGA_24_-3′) or with 5′NCR of CHIKV genome were generated by in vitro-transcription. Following incubation with the CHIKV-nsP2T at 37°C for 30 min, products were analyzed by TLC. 5′-[γ-^32^P]-RNA or 5′-[α-^32^P]-RNA substrates were separately incubated with shrimp alkaline phosphatase (SAP) to see the specificity of CHIKV-nsP2T for phosphate group at the 5′-end of the RNA substrate. As expected, SAP could remove ^32^P moiety from both RNA substrates, but CHIKV-nsP2T showed no release of label from 5′-α-^32^P-RNA. CHIKV-nsP2T released labeled 5′-phosphate group from 5′-γ-^32^P labeled RNA indicating that CHIKV-nsP2T hydrolyzes only γ-β-triphosphate bond and unlike SAP does not have a general phosphohydrolase activity that would also remove the β- and α- phosphate groups (Figure 5a). This suggested that CHIKV-nsP2T has 5′-RNA-triphosphatase (RTPase) activity. Extent of hydrolysis of both CHIKV 5′-NCR and non specific RNA oligo substrates was comparable (Figure 5b). Thus, for the further characterization of 5′-RNA-triphosphatase activity, non-specific RNA substrate was used.

**Figure 6:**
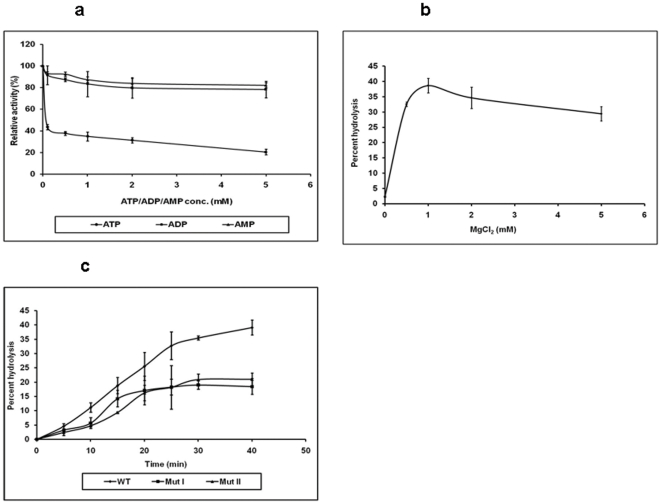
Effect of different conditions on the RNA 5′-triphosphatase activity of CHIKV-nsP2T. Effect of AMP, ADP and ATP on RTPase activity: CHIKV-nsP2T was incubated with 5′-[γ-^32^P]-RNA at 37°C for 30 min in presence of different concentrations of AMP/ADP/ATP independently and products were analyzed by TLC. Activity of CHIKV-nsP2T without AMP/ADP/ATP was taken as 100% and the percent activity of each reaction was calculated separately for each reaction. The effect of MgCl_2_ on RTPase activity: CHIKV-nsP2T was incubated with 5′-[γ-^32^P]-RNA at 37°C for 30 min in presence of different concentrations of MgCl_2_ (0 -5.0 mM). Released radiolabel [^32^Pi] was quantitated for three independent experiments and mean values were plotted. RTPase activity of nsP2 mutants: CHIKV-nsP2T wild type, mut I and mut II proteins were incubated with 5′-[γ-^32^P]-RNA at 37°C. Aliquots were removed at different time points (5, 10, 15, 20, 25, 30 and 40 min) and analyzed. Released radiolabel [^32^Pi] was quantitated for three independent experiments and mean values were plotted.

To see the effect of ATP, ADP and AMP on RNA triphosphatase activity of CHIKV-nsP2T, RNA was incubated in presence of different concentrations of ATP, ADP or AMP. ATP showed 80% inhibition of RTPase, activity indicating that ATP and RNA shared the substrate binding site. ADP and AMP did not affect the RTPase activity even at 5 mM concentration (Figure 6a), suggesting that the 5′-terminal γ-phosphate group in the substrate is the major determinant for competition. These data led us to conclude that CHIKV-nsP2T NTPase and RNA 5′-triphosphatase activities have a common active site and 5′-terminal γ- and β-phosphate groups interact with the NTPase/RNA 5′-triphosphatase activity domain of CHIKV-nsP2T.

As seen in the earlier experiments, NTPase activity of CHIKV-nsP2T was completely dependent on Mg^2+^. To see the effect of Mg^2+^ on RTPase activity of the enzyme, reactions were carried out in presence of different concentrations of MgCl_2_ (0 to 5.0 mM). Enzyme showed 2-3% release of phosphate in absence of Mg^2+^. There was significant increase in the percent phosphate release (up to 40%) in presence of 1 mM MgCl_2_ (Figure 6b). This confirmed requirement of Mg^2+^ ions also for the RNA triphosphatase activity.

Mutations in the Walker A motif, nsP2 mut I and Walker B motif, nsP2 mut II resulted in a significant loss of ATPase activity. The conserved lysine residue in Walker A motif A is important for interaction with β, γ phosphate of NTP and hence mutations in this region directly affected NTPase activity. To find out the effects of the mutations on the RTPase activity, the mutant proteins were incubated with 5′-γ-^32^P RNA substrate. Both mutants showed 50% reduction in the percent phosphate release (Figure 6c). Overall, effects of mutations on NTPase and RTPase activities were similar, suggesting that the NTPase and RTPase activities shared a common reaction mechanism.

## Discussion

Protease activity of CHIKV nsP2 has already been demonstrated with C-terminal protease domain containing 422–799 a.a. long protein [10]. In the present study, to test the enzymatic activities associated with the N-terminal NTPase/helicase domain of CHIKV nsP2, 465 a.a. (166–630 a.a. of the full-length nsP2), was expressed as a MBP fusion protein in E.coli (Figure 1a, b). Optimal conditions for the NTPase activity of CHIKV-nsP2T were similar to earlier reported helicase-like proteins from positive sense ssRNA viruses. CHIKV-nsP2T NTPase activity was completely dependent on Mg^2+^ ions (Figure 2c) as reported for Alphaviruses [13].

CHIKV-nsP2T could hydrolyze all NTPs except dTTP. Extent of hydrolysis of CTP, dCTP and UTP was very low, while there was a moderate level hydrolysis of dATP, GTP and dGTP. ATP appeared as the best substrate of CHIKV-nsP2T from all the NTPs. This pattern of substrate preference was similar to SFV [14], Flaviviruses [15], [16], Coronaviruses [17], and Hepatitis E Virus [12], which are general NTPases and can utilize any NTP.

CHIKV-nsP2T NTPase activity was not stimulated by nucleic acids. The ssRNA binding motif (Ia) was found to be highly conserved in CHIKV helicase domain (Figure 1a) however enzyme did not show any enhancement with RNA. It has been suggested that the highly conserved Ia ssRNA binding motif of SF1 helicases helps in stimulating NTPase activity [18], [19]. SF1 helicase superfamily has members showing different levels of enhancements in their NTPase activities in response to RNA binding. The superfamily has members like turnip yellow mosaic virus [20], rubellavirus [21], semliki forest virus [14] and hepatitis E virus [12] showing marginal enhancement in the activity (1.5 to 2 fold) and members like SARS coronavirus [17], human coronavirus 229E (HCo-229E) [22], [23], and equine arteritis virus [24] showing 15- to 20-fold enhancement in the activity. CHIKV-nsP2 belongs to the first category.

CHIKV-nsP2T showed no detectable strand separation activity even after using higher amounts of protein in the unwinding assay (1000ng/reaction). It is possible that as seen in many positive strand RNA viruses with helicase-like proteins having only NTPase activity without any unwinding ability [25], CHIKV-nsP2 is just an NTPase. Other possibility is that unwinding activity of CHIKV-nsP2 is dependent on the protease domain. Helicase motifs identified until now are actually characteristic of NTP-dependent nucleic acid translocases which are capable of moving unidirectionally along a single or double stranded nucleic acid. Extra domains in addition to core enzyme (need not be from the same protein) may be necessary for conferring strand separation activity to the enzyme. As discussed above, CHIKV nsP2 may also need help of some other CHIKV nonstructural protein to carry out the strand separation function.

Point mutations in Walker A and Walker B motifs of CHIKV-nsP2T resulted in 5 fold decrease in the ATPase activity as compared to wild type protein. Walker A motif has a consensus of XGX-AGXGKT in SF1 helicases [26]. The lysine residue is responsible for binding to the β- and γ- phosphates of NTP-Mg^2+^ complexes and mutations in this residue result in significant loss of the NTPase activity [27]. Complete inhibition of genome replication was observed when Walker A motif was altered in SFV infectious cDNA clone [28]. The conserved D residue in the Walker B motif (motif II, DEXX) has been shown to interact with Mg^2+^ [29] and also results in the significant loss of NTPase activity in Alphaviruses.

Alphaviruses possess a novel RNA capping mechanism, where GTP is first methylated, forms a covalent m^7^GMP intermediate with nsP1and then gets transferred on to RNA resulting in G_0_ cap structure. Methyltransferase and guanylyltransferase activities required to carry out these reactions have been demonstrated to be nsP1 associated [30]. There is prior step of removing 5′ γ- phosphate of the nascent RNA and nsP2 has been identified as the RNA triphosphatase for both SFV and SINV [6]. On testing CHIKV-nsP2T for RNA 5′-triphosphatase (RTPase) activity, it released ^32^P only from the 5′-[γ-^32^P] RNA and not from the 5′-[α-^32^P] RNA substrate showing specificity for the γ - β -triphosphate bond (Figure 5a) confirming CHIKV-nsP2T having RTPase activity. Release of the 5′-phosphate group from the nonspecific RNA oligonucleotide substrate and that from the CHIKV 5′-NCR were equally efficient. This showed that CHIKV-nsP2T 5′-RTPase has no sequence specificity for RNA substrates. RNA-5′ triphosphatase activity of the mutant proteins showed significant inhibition (Figure 6c) suggesting that the NTPase and RTPase activities shared a common reaction mechanism. The effective inhibition of RTPase with 0.1 mM ATP and not with ADP and AMP further confirmed that the enzyme binds by means of triphosphorylated nucleotide. In summary, CHIKV-nsP2T has both NTPase and RNA 5′-triphosphatase activities. It could hydrolyze all NTPs except dTTP and showed better efficiency for ATP, dATP, GTP and dGTP hydrolysis. ATP was the most preferred substrate by the enzyme. RTPase activity was completely inhibited by ATP showing sharing of the binding motif by NTP and RNA. NTPase and RTPase activities were completely dependent on Mg^2+^ ions. Walker A and Walker B mutant proteins showed significant reduction in both enzymatic activities confirming sharing of functional domains for these activities. This is the first report showing CHIKV-nsP2 associated NTPase and RNA 5′-triphosphatase activities. Considering importance of these functions during alphavirus replication, helicase could serve as a potential target for antivirals.

## Materials and Methods

### Virus and cloning

Viral RNA was isolated from infected C6/36 cell culture supernatant (Virus: Andhra Pradesh strain, isolated during 2006 outbreak in India, Genbank accession number: EF027134.1) using QIAamp viral mini kit (Qiagen, Germany) as per the manufacturer's instructions. Purified RNA was reverse-transcribed using Superscript II reverse transcriptase (Invitrogen). Truncated nsP2 region (2161–3552 nt, spanning 166–630 a. a. of the CHIKV nsP2) was PCR amplified using Pfx polymerase (Invitrogen) and cloned in NdeI/BamHI sites of pMal-5cX vector (New England BioLabs) (NEB).

### Protein purification

The recombinant vector pMal-5cX-nsP2 was transformed into E.coli BL21 (DE3)/pLysS host cells for protein expression. E.coli cells were also transformed with pMal-5cX vector. Protein induction was done with 1.0 mM IPTG (isopropyl-β-D-thiogalactopyranoside) for 4 h at 25°C. The fusion protein (fused with maltose-binding protein) (MBP) was purified from bacterial culture pellets by using amylose resin column (NEB). A parallel purification of MBP was carried out from the empty vector transformed cells to obtain control fraction which was used to test absence of contaminating enzymatic activities from E.coli in the purified protein. Briefly, 100 ml culture equivalent cell pellet was lysed by using 10 mg lysozyme (Sigma) in binding buffer (20 mM HEPES, pH 7.5, 150 mM NaCl, 10 mM β-mercaptoethanol). Cell lysate was spun at 10,000 g for 30 min, filtered through 0.45 µm syringe filter (Millipore) and loaded on to the affinity column equilibrated with binding buffer. Elution of the protein was performed using binding buffer containing 10 mM Maltose (Sigma). Collected fractions were analyzed on 10% SDS-PAGE and fractions containing protein of the expected size were combined and concentrated by using Amicon membrane columns (cutoff, 50 kDa; Millipore). The protein was further purified by gel filtration chromatography (Sephacryl HR100, CV-120 ml; Amersham Biosciences) by using an Akta Basic 100 HPLC system (Amersham Pharmacia, USA). Fractions were analyzed on 10% SDS-PAGE. Fractions containing purified protein were combined, concentrated by using an Amicon membrane column and buffer was exchanged to 50 mM HEPES (pH 7.0). Glycerol and DTT were added to the final concentrations of 20% and 2 mM respectively and stored at −70°C in aliquots. Western blot analysis was done using anti-MBP polyclonal antibodies (NEB).

### Site directed mutagenesis

To modify underlined amino acid residues from Walker A motif- GKS to GAS and Walker B motif- DEAF to AAAF in the CHIKV-nsP2T protein, site directed mutagenesis was carried out using pMal-5cX-nsP2 clone as a template and QuickChange XL Site-Directed mutagenesis Kit (Stratagene, La Jolla, CA) as per the manufacturers' instructions. Clones were confirmed by sequencing and transformed into E.coli BL21 (DE3) pLysS cells. Procedure for the protein induction and purification was same as described above.

### NTPase assays

#### Colorimetric assays

NTPase assays were performed by measuring phosphate release using a colorimetric method based on complex formation with malachite green and molybdate as described earlier [12]. Briefly, in a 50 µl reaction, 1 nM of CHIKV-nsP2T (4.75 ng/50 µl reaction), 50 mM MOPS (pH 7.25), 1 mM MgCl_2_, 0.05 mg of bovine serum albumin (BSA) per ml, 2 mM DTT, 1 mM NTP were added and incubated at 37°C for 30 min. Reactions were performed in 96 well plate. When indicated, various 19 nt oligonucleotides [either poly- (U), (A), (C), (G) RNAs or poly- (T), (A), (G), (C) DNAs] were included in the reaction. Three independent sets of experiments were carried out to determine the kinetic parameters. Lineweaver-Burk plots (1/v vs. 1/s) were drawn for each substrate using Enzyme Kinetic Module of the Sigma Plot and the *K_m_* and *k_cat_* values were determined.

#### Thin layer chromatography

Reaction was carried out in 20 µl volume containing 1 nM CHIKV-nsP2T, 50mM MOPS (pH 7.25), 1 mM MgCl_2_, 0.05 mg of BSA per ml, 2 mM DTT, 1 mM ATP, 1 µCi of [γ-^32^P] ATP, (3000 Ci/mmol, BRIT, India). Reactions were incubated at 37°C for 30 min and then terminated by the addition of EDTA to the final concentration of 20 mM. Analysis of the hydrolysis products was done by spotting 1 µl reaction mixture onto a polyethyleneimine-cellulose thin layer chromatography plate (TLC) (Merck), and separation was carried out using 0.375 M Potassium phosphate (pH 3.5) as the mobile phase. Plate was air dried and exposed to X-ray film.

### Preparation of helicase substrates

RNA substrates were prepared as described earlier [12].

### Strand displacement assays

The 20 µl unwinding reaction containing 50 mM MOPS (pH 7.25), 1 mM MgCl_2_, 0.05 mg of BSA per ml, 2 mM DTT, 1 mM ATP, 1 pmol labeled RNA duplex, 1 nM CHIKV-nsP2T was incubated at 37°C, for 2 h. Reaction was terminated using 0.2% SDS and 20 mM EDTA (final conc.) followed by proteinase K treatment for 10 min, at 37°C. Products were resolved on 20% PAGE and gels were processed for autoradiography.

RNA triphosphatase assay.

Two RNA substrates, with a nonspecific sequence (5′-GGGA_24_-3′) and 5′ non-coding region (5′-NCR) (27 bases) of CHIKV genome (Andhra Pradesh strain, Genbank accession number: EF027134.1) with either α- or γ-^32^P 5′-end label were generated by in vitro transcription in presence of 50 µCi of either [α-^32^P]GTP or [γ-^32^P]GTP (each 3200 Ci/mmol, BRIT, India) respectively as described earlier [13] The double stranded DNA templates used for transcription reactions were prepared by annealing following pairs of oligonucleotides- A) nonspecific RNA:

5′-AAAATAATACGACTCACTATAGGG (A)_24-_3′ (T7 promoter sequence underlined) and 5′-(T)_24_CCCTATAGTGAGTCGTATTATTTT-3′, B) 5′-NCR: 5′-AAAATAATACGACTCACTATAGTAGCCTACCAGTTTCTTACTGCTCTA-3′ and 5′-TAGAGCAGTAAGAAACTGCTAGGCTACTATAGTGAGTCGTATTATTTT-3′.

The RNAs were separated from unincorporated radioactive nucleotides by two rounds of gel filtration through Sephadex-G50 columns.

RNA triphosphatase assay was carried out in 20 µl reaction containing 50 mM MOPS (pH 7.25), 1 mM MgCl_2_, 0.05 mg of BSA per ml, 2 mM DTT, 1 nmol of CHIKV-nsP2T, 100 nmol of 5′-γ-^32^P or 5′-α-^32^P labeled RNA. Following incubation at 37°C for 30 min, products were analyzed by TLC. For quantitation of amount of Pi released from RNA, spots from TLCs were cut, put in vials along with scintillation fluid and counts were taken in the Packard Tri-carb 2810TR liquid scintillation counter.
